# Multilayer biocomposite vegan leather materials derived from vegetable-tanned fungal biomass cultivated on food waste

**DOI:** 10.1038/s41598-025-98361-w

**Published:** 2025-05-02

**Authors:** E. R. Kanishka B. Wijayarathna, Sofie E. Svensson, Taner Sar, Akram Zamani

**Affiliations:** Swedish Centre for Resource Recovery, University of Borås, 501 90 Borås, Sweden

**Keywords:** Food waste, Filamentous fungi, *Rhizopus delemar*, Fungal materials, Vegan leather, Prototype, Bioinspired materials, Bioinspired materials

## Abstract

Despite being considered a premium material, leather poses both environmental and ethical issues. Thus, sustainable alternatives such as vegan leather are in high demand. Therefore, in this study, we aimed to produce vegan leather using vegetable tannins and fungi grown on bread waste. Fungal cultivation was carried out in a bubble column bioreactor using nutrients extracted from bread as substrate. To obtain tanned biomass, the biomass was subjected to vegetable tanning (using Tara, Myrobalan, Chestnut, and Indusol ATO tannins). A mild alkali treatment isolated the fibrous cell wall material from fungal biomass. Different composite sheets were prepared by wet-laying the tanned biomass and cell wall material and placing them in a multilayer arrangement. The composites were post-treated with glycerol and a bio-based binder to improve their mechanical properties. Myrobalan-tanned biomass composites after glycerol and bio-based binder post-treatments had the highest flexibility of 14.8% elongation at break, and Tara-tanned biomass composites had the highest tensile strength of 20.5 MPa. Ashby’s chart demonstrates the relationship between the sheets produced and natural leather. SEM was used to demonstrate the softer and smoother morphologies of the Chestnut and Indusol ATO-tanned composite sheets after post-treatment. Overall, this study presents multilayer fungal biocomposites as a promising vegan alternative leather.

## Introduction

Leathers traditionally produced using animal hides are considered one of the oldest historical materials^1,2^. Today, leather products are considered luxury goods, with a market value of USD 394 billion in 2020^3^, and earning 50 billion in turnover annually^4^. Nevertheless, in the recent past, the leather industry has been accused of generating environmental and ethical issues such as animal rights concerns, poor working environments, water pollution, and the use of toxic chemicals^3–5^.

In response to these issues, alternative materials, called vegan leather, have been developed from non-animal sources to replace natural leather. Vegan leather has been a fashion trend for several years but is frequently questioned regarding sustainability concerns despite its cruelty-free production^6^. Moreover, property-wise vegan leather has always fallen behind natural leather because the natural structure of animal hides has a multiscale arrangement with a tightness gradient to perform load-bearing functions^7^. On the other hand, over the past five to six years, researchers and companies have been working on developing better alternative leather materials that fulfil both product properties and sustainability requirements. As a result, petroleum-based, plant-based, and microbial-based vegan leather have been produced, and some materials have been commercialised by high-end fashion brands^6^. Petroleum-based vegan leather is mostly made of polyvinyl chloride (PVC) or polyurethane (PUR) using textile backing, and the material has the feel and appearance of natural leather^2,7^. Due to the environmental issues and non-biodegradability of both PVC and PUR, consumers have rebutted these materials. Moreover, Meyer et al.^7^ questioned the use of polyurethane (PUR) as a coating to either protect or reinforce plant-based vegan leather.

Microbial-based leather materials are mainly produced from bacteria and fungi, and fungal-based vegan leathers such as Mylo™, Forager™ Hides, and Reishi™ have gained much attention from fashion brands over the past half-decade. These commercialised mycelium leathers are produced mainly by solid state fermentation^8,9^ using agricultural residues, however the details are kept as trade secrets thus most of the important information is not available to the public or the research world^10^. To produce some of those materials genetically modified fungal strains are used while polyurethane (PU) coatings are used as a protection layer in some other materials^2,3,10^. Although fungal leather materials are far better than natural leather in environmental and ethical aspects, it is still necessary to find an alternative to PU-based coatings and make their production even more benign. In our previous study, the production of materials with leather-like properties using the filamentous fungus *Rhizopus delemar* cultivated on bread waste was successfully demonstrated in our previous study^11^. *Rhizopus delemar* is a fast-growing fungus which can grow on minimum nutrients^12^. The materials produced were single-layer and thin, with a rough handfeel. Moreover, the cell wall of *R. delemar* contains naturally synthesised chitin and chitosan^13,14^ which can be extracted and utilised to produce stronger materials, such as monofilaments, to be used as textile fibres^15–17^. Benedikt Maria Köhnlein et al.^18^ have shown the production of stronger wet-laid sheets (18.1 MPa^18^) compared to the single-layer leather sheets (6.9 MPa^11^) using cell wall materials that mainly contain chitin and chitosan from the same fungus *R. delemar*.

Building upon previous studies, this study explores a novel multilayer composite structure by integrating tannin-treated fungal biomass with a chitin- and chitosan-rich cell wall fraction. We hypothesised that a multilayer composite structure composed of tannin-treated fungal biomass reinforced with fungal cell wall material would mimic the cross-sectional arrangement of natural leather. Four different vegetable tannins were tested on fungal biomass for the first time, and alkali treatment was done on biomass to isolate the chitin and chitosan-rich fungal cell wall fraction. Wet-laid sheets were made from different samples of tanned biomass and the isolated cell wall fraction and then combined in a multilayer sandwich composite formation. Glycerol and bio-based binder post-treatments were performed on the sheets to improve their properties. The mechanical properties of the produced materials were compared with those of natural leather using Ashby plots^19^. Finally, the workability of the materials was demonstrated by fabricating a prototype. To the best of our knowledge, a complete fungal-based multilayer composite sheet that can introduce different properties from different layers mimicking natural leather^7^ has not been produced or tested before. Valorising food waste by growing fungi and creating materials with leather-like properties and proven workability highlights the potential of entirely bio-based composites for sustainable vegan leather.

## Materials and methods

### Microorganism and materials

Mucoromycete fungus (zygomycetes according to the old classification) *Rhizopus delemar* CBS 145,940 (Centraalbureau voor Schimmelcultures, Utrecht, The Netherlands), isolated from tempeh, was used in this study^13^. Commercial vegetable tannins i.e.: Tara, Myrobalan, Chestnut, and Indusol ATO (Quebracho extracts), were kindly supplied by Silvateam S. p. a., Italy. The bio-based binder, OrganoClick Lotus®, was kindly provided by OrganoClick AB (Sweden). Agar, peptone, α-amylase (heat-stable, enzyme activity 20,000–60,000 U/mL), and sodium hydroxide pallets were purchased from Sigma-Aldrich. Glucose (anhydrous), glycerol (purity > 99.7%), and hydrochloric acid (6.0 N) were purchased from VVR Chemical. The unsold waste bread was collected from nearby supermarkets of ICA Gruppen AB, Sweden. The bread was broken into small pieces by hand, dried at room temperature for two–three days, crushed into a powder with a particle size of ≤ 3 mm using a rotary dry mill (M 100, Retsch Technology GmbH, Germany), and stored at room temperature until use.

### Bread hydrolysis

In our previous study, fungal cultivation was done on suspended bread particles^11^. There it was observed that the brown crust in baked products caused by the Maillard reaction, caramelisation^20^, and other particles available in bread (seeds, nuts, and fruits) were not consumed and entangled in the final harvested biomass^11^. These entanglements cause problems in the final material properties. To eliminate this, the bread was treated with α-amylase to hydrolyse and solubilise starch, and the filtered liquid was used as the substrate for fungal cultivation. Bread hydrolysis was performed in batches of 45 l with 9 kg (20% w/w) of ground bread using a brewing kettle (Digiboil Kegland, Australia) at 80 °C and pH 6.9 for 2 h. During the enzymatic treatment, the slurry was mixed every 15 min using an industrial wisp (Robot-Coupe, France). After hydrolysis, the liquid fraction was filtered using 210 µm nylon filter bags (Brew bags, USA) and stored in clean containers at 4 °C until use.

### Fungal cultivation

Fungal cultivation was performed in four steps: agar plates, 500 ml Erlenmeyer flasks, a 26 l bubble column bioreactor, and a 1300 l bubble column bioreactor. For the agar plates, suspensions were prepared with 17.0 g/l agar, 20 g/l glucose, and 4 g/l peptone. The pH of the solution was adjusted to 5.5 and sterilised at 121 °C for 20 min in an autoclave (VX-95, Systec, Linden, Germany). The sterilised solution was cooled, poured into sterile petri dishes, and allowed to harden. 0.1 ml of fungal spores (containing 14 × 10^6^ spores/ml, as measured using a Bürker counting chamber), prepared aseptically by adding 20 ml of sterile water to a previously prepared fungal agar plate and scraping the spores into the liquid using a sterile scraper, was used to inoculate each agar plate. After inoculation, the plates were incubated at 30 °C. After 3–4 days, the plates were removed, covered with parafilm, and stored at 4 °C until use.

Liquid submerged fermentation was used for the next three scaled-up cultivation steps. The liquid fraction from the hydrolysed bread was used as the substrate. The solid content of the liquid fraction was 14.75 ± 0.5%, and it was diluted to 4% with water. For all the liquid culture substrates, 1 g/l yeast extract was added to the diluted bread hydrolysate (4%), and the pH was adjusted to 5.5 before sterilisation. Liquid submerged cultivation was started with two 500 ml Erlenmeyer flask cultivations with 200 ml of liquid substrate (prepared as mentioned before). The flasks were sterilised in an autoclave (VX-95, Systec, Linden, Germany) at 121 °C for 20 min. The inoculation was prepared with 4 ml of spore suspension, and cultivation was continued for 24 h. After 24 h, the culture broth was used as the inoculum for the 26 L bubble column bioreactor. The 26 l bioreactor was sterilised in situ with steam at 121 °C for 20 min. The liquid substrate 20 l was prepared as described previously by diluting the bread hydrolysis liquid fraction to 4%, adding 1 g/l yeast extract, and sterilising it at 121 °C for 20 min using the same autoclave. Cultivation in the 26 l bubble column bioreactor was also conducted for 24 h, and then the broth was used as the inoculum for the 1300 l bubble column bioreactor. For the 1300 l bioreactor, 1000 l substrate was used. The empty reactor was sterilised in situ with steam at 121 °C for 20 min. The substrate was then filled and sterilised in the reactor at 121 °C for 20 min. After cooling, the reactor was ready to inoculate the inoculum obtained from the 26 l bubble column reactor. The final cultivation step was 48 h. The pH was manually adjusted to 5.5 using NaOH. After 48 h, the biomass was harvested using 210 microns nylon filter bags (Brew bags, USA). The biomass was washed twice with water and stored in plastic bags at – 18 °C until use. This biomass was subjected to different pre-treatments to prepare the materials required for wet-laid sheet-forming.

### Fungal biomass tanning

Tanning of fungal biomass was performed using the method described in detail in our previous work^11^, and is briefly described here. First, the biomass suspension in water was ground using an ultrafine grinder (Masuko, Japan) at 2700 ± 50 rpm with an open 50 µm gap between the grinder stones (MK E6–46 DD), and then the pH was adjusted to 3.5 using HCl. Tannin was added at a concentration of 15 g/l and tanning fixation was continued for eight days at 25 °C with mechanical stirring^21^. Instead of using one tannin type in the previous study, four different vegetable tannins, Tara, Myrobalan, Chestnut, and Indusol ATO, were used. After the tannin treatment, the unfixed tannins were washed and purged. Tanned biomass was stored at 4 °C until further use.

### Alkali treatment on fungal biomass and preparation of hydrogel using the fibrous cell wall material

Alkali treatment was performed using NaOH solution and heating. First, the fungal biomass was suspended in water and homogenised using an ultrafine grinder (Masuko, Japan) using MKE6-46 DD grinding stones with an open 50 µm gap size and 2700 ± 50 rpm. Grinding was done by passing the biomass suspension through the grinder twice. Then a NaOH solution was added to the biomass suspension to adjust the final concentration of NaOH and biomass to 0.1 M and 30 g dry biomass per litre of the whole suspension, respectively. After thorough mixing, the suspension was heat-treated at 121 °C for 20 min in an autoclave (VX-95, Systec, Linden, Germany). Fibrous cell wall material was recovered as alkali-insoluble material by filtering and washing the insoluble fraction from the alkali treatment until a neutral pH was reached. To eliminate the clumps of the recovered material, a homogenised hydrogel was prepared. The hydrogel was prepared by adding lactic acid to the recovered fibrous cell wall material and the final pH of the hydrogel was adjusted to 3 ± 0.5^22^.

### Wet-laying of tanned biomass and preparation of multilayer biocomposites

**Fig. 1:**
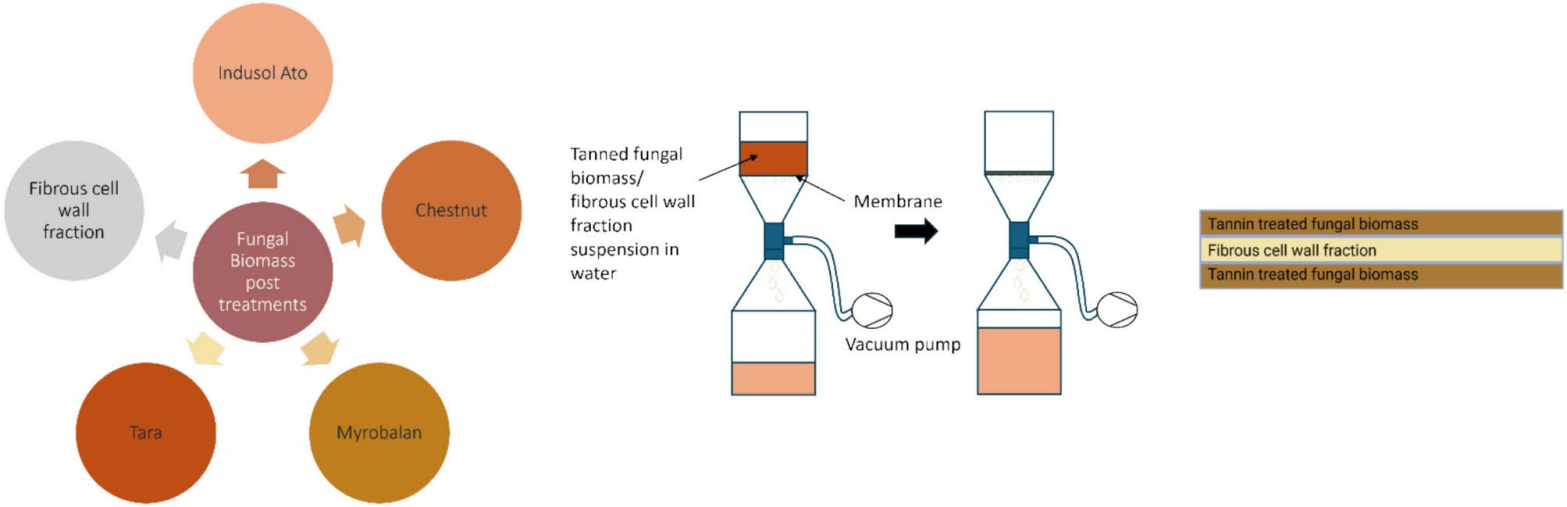
The schematic on the composite sheets production including different post-treatments on fungal biomass, the wet-laying process used to prepare the composite leather like materials and the multilayer arrangement in the composite sheets.

Wet-laying of tanned biomass and the hydrogel obtained from fungal cell wall material was done and multilayer composites were prepared by combining wet-laid sheets. Wet-laid sheets of 100 mm diameter were made using a vacuum funnel (Sterlitech, USA) and a 30 µm pore size nylon membrane (Spectra Mesh® woven filters, Nylon, Thermo Fisher Scientific, USA)^11^. The sheets were dewatered using a blotting paper. Composite sheets were prepared by sandwiching one cell wall hydrogel sheet with two tannin-treated biomass sheets, as shown in Fig. 1. The sandwiched composite was pressed at 12 kN for 5 min using an oil benchtop press (Rondol Technology, UK). Finally, the sheets were dried at room temperature by stacking one on top of the other on the plastic sheets using plastic rings. To reduce shrinkage during drying, the stack was held at a weight of approximately 3 kg from the top.

### Post-treatments of the sheets with glycerol and bio-based binder

To enhance the mechanical properties of the dried sheets, glycerol and bio-based binder post-treatments were performed. The process was explained in detail in our previous study^11^, and is described briefly with alterations. The dried sheets were soaked in a 20% (v/v) glycerol solution for 30 min. The sheets were then dewatered using blotting papers, followed by drying with the same setup described in Section 2.6 to maintain the material dimensions. For the bio-based binder treatment, the sheets after glycerol treatment were applied with the bio-based binder using a regular paintbrush. The application was performed on both sides, and the sheets were dried at room temperature on plastic rings without any support, as the sheets did not shrink after the post-treatment according to our preliminary observations^11^. After the post-treatments, sample nomenclature was performed according to Table 1.

**Table 1:** Sample nomenclature after the post-treatments.

Sample	Description
Tara S	Tara tanned single layer
Tara C	Tara tanned sandwich composite with AIM
Tara C G	Tara tanned sandwich composite with AIM post-treated with glycerol
Tara C GB	Tara tanned sandwich composite with AIM post-treated with glycerol and bio-based binder
Myrobalan S	Myrobalan tanned single layer
Myrobalan C	Myrobalan tanned sandwich composite with AIM
Myrobalan C G	Myrobalan tanned sandwich composite with AIM post-treated with glycerol
Myrobalan C GB	Myrobalan tanned sandwich composite with AIM post-treated with glycerol and bio-based binder
Chestnut S	Chestnut tanned single layer
Chestnut C	Chestnut tanned sandwich composite with AIM
Chestnut C G	Chestnut tanned sandwich composite with AIM post-treated with glycerol
Chestnut C GB	Chestnut tanned sandwich composite with AIM post-treated with glycerol and bio-based binder
Indusol ATO S	Indusol ATO tanned single layer
Indusol ATO C	Indusol ATO tanned sandwich composite with AIM
Indusol ATO C G	Indusol ATO tanned sandwich composite with AIM post-treated with glycerol
Indusol ATO C GB	Indusol ATO tanned sandwich composite with AIM post-treated with glycerol and bio-based binder

### Fluidscope™ scanning of tanned biomass (oCelloScope)

To measure the fungal hyphae diameter before and after the tanning process, Fluidscope™ scanning analysis was done using an oCelloScope (BioScience Solutions, Denmark). The biomass suspensions were diluted 100 times, and 1 ml of the diluted sample was added to one well of a 24 well plate (Sigma-Aldrich, USA) to obtain clear images from the microscope. The number of images was set to 20, with an illumination time of 2 ms. The imaging distance was 4.9 µm and the focus was automatically adjusted by the instrument depending on the sample. Fifty fungal hyphal diameter measurements were obtained for each sample, and the results are shown as the average ± standard deviation.

### Fourier transform infrared spectroscopy

The tanned biomass and untreated biomass were analysed using Fourier transform infrared spectroscopy (FTIR, Nicolet iS10, Thermo Fisher Scientific, USA) to investigate the reaction between the fungal biomass and tannin. To remove excess moisture, the samples were kept in a desiccator for 24 h before the test. The spectra were corrected with a background spectrum to eliminate the absorption due to atmospheric noise. The raw data obtained were normalised and plotted using Origin software (Origin 2022 (64-bit) SR1 9.9.0.225, Academic).

### Mechanical properties (tensile strength and elongation % at break)

The mechanical properties of the produced sheets were analysed by performing a tensile test. The thickness of the sheets was measured at five points using a gauge (Mitutoyo, Japan), and the average was used as the input for the tensile test software (Horizon, Tinius Olsen, USA). According to ISO 527-2 (2012), test specimens of the type 5A dog bone shape were cut from the sheets using a press knife (Elastocon, Sweden). The cut specimens were conditioned in a standard atmosphere at 23 ± 2 °C and 50 ± 5% relative humidity for 24 h before the test^23^. The test was performed using a tensile testing machine (HK10, Tinius Olsen, USA) with a load cell of 100 N, the length between the clampers was fixed at 25 mm, and the test speed was 1 mm/min. The values for each property were obtained using the same software and are presented as mean ± standard deviation of minimum three tests for each sample in Table 2.

**Table 2:** Fungal hyphae diameters before and after each tannin treatment are shown as average ± standard deviation of 50 measurements.

Sample	Hyphae diameter
Untreated	7.01 ± 1.42
Tara	9.52 ± 2.15
Myrobalan	9.78 ± 2.42
Chestnut	8.52 ± 2.21
Indusol ATO	9.78 ± 2.48

### Lightfastness test

Lightfastness or colour fading due to light was tested using Xenotest 440 (Atlas Material Testing Technology, USA). The test was done according to ISO 105-B02:2014 exposure cycle A1, with a small modification. The black standard temperature was adjusted to 47 ± 5 °C instead of 47 ± 3 °C, owing to instrument limitations. The other parameters were maintained according to the standard. The test was stopped when the blue wool reference 2 became equal to grey scale grade 3 (Grey Scale for assessing Change in Colour—ISO 105 A02:1993—James Heal, USA) observed under D65 (artificial daylight) illuminant. The total runtime was 24 h. The samples were analysed according to ISO 105-A05:1996 using a spectrophotometre (Datacolor 500, USA) by testing five samples each from the reference (unexposed sample) and test specimen (exposed sample) using a CIE illuminant D65 and 10° observer. The results are shown in grey scale rating for colour change (GS_c_) mentioned in ISO 105-A05:1996, obtained using Datacolor TOOLS Plus software.

### Abrasion resistance test (martindale method)

The abrasion resistance test was carried out using Nu-Martindale 404 Abrasion & Pilling tester (James Heal, UK) by combining ISO 12947-4:1998 and ISO 17076-2:2011. This combination was used because of the limitations of the sample size. The ball plate with a diameter of 120 mm, recommended in ISO 17076-2:2011, could not be utilised because the original sample prepared had a diameter of 100 mm. Therefore, the test was done according to ISO 12947-4:1998 using a sample with a diameter of 38 mm, a loading piece of 12 kN, and SM25 Wool Abradant Fabric (SDL Atlas, USA) as the standard abrading material. The test was initiated with 100 rubs, and the samples were visually assessed to count the number of places with complete breakdown. Subsequently, the test was continued at 100 s intervals until four or more breakdown places appeared. The test was carried out in duplicate, and the results are shown as the number of rubs required to reach four finish breakdowns. An optical microscope (Nikon Eclipse LV150NL—Transmission Microscope) was used for assessment, and image collection was performed using the NIS-Elements BR 5,41,01 64-bit software.

### Surface morphology and cross-sectional observations from microscopy

The surface morphologies of the sheets were observed using scanning electron microscopy (SEM). The samples were prepared by attaching them to carbon tape and coating them with gold. Ultra-high-resolution field-emission scanning electron microscopy (FE-SEM) (Zeiss, Sigma, Germany) was used to acquire the images. Photomicrographs at 300  magnification with 25.00 kV accelerating voltage were obtained and presented in the results (Fig. 1).

To observe the cross-sectional view of the multilayer composite arrangement, optical microscopy observations were used. Nikon Eclipse LV150NL—Transmission Microscope and NIS-Elements BR 5,41,01 64-bit software were used to obtain images.

### Material property analysis with Ashby’s charts

A material property analysis was performed to compare the sheets produced with natural leather. The computerised material selection chart software developed by Prof. Mike Ashby is a widely accepted method^19,24,25^ for material comparison. Granta Edupack 2021 R2 version: 21.2.0 (Ansys Inc. USA) was used to compare the acquired properties of the fungal sheets with the properties of natural leather saved in the software database. To perform the material comparison, the tensile strength, the elongation % at break and young’s modulus were plotted against the density using the Level 1 material database of the software (Fig. 6A–C).

### Statistical data analysis

All data analyses were performed using Minitab 21 (Minitab® 21.1.1). The significance between two compared values was tested using 2 sample t test with 95% probability. Unless otherwise mentioned, the number of data replicates was either equal to or greater than three in all tests. The raw data supporting the results is provided in the supplementary information file. 

## Results and discussion

Natural leather is a historical and excellent product; however, owing to the environmental and ethical issues of the leather industry, the fashion world is constantly moving towards sustainable vegan leather alternatives. Therefore, the aim of this article is to present a completely bio-based material made of fungi that can be used as a substitute for natural leather. Nutrient recovery from bread waste was performed, and the fungus was cultivated on it. The fungal biomass was then subjected to two different treatments. One was to use vegetable tannins to stabilise proteins. The other was an alkali treatment using dilute sodium hydroxide to isolate the chitin- and chitosan-rich cell wall as an alkali-insoluble material (AIM). Wet-laid sheets were produced using both tanned biomass and AIM and then laid in a sandwiched composite arrangement. The produced multilayer biocomposites were subjected to glycerol and bio-based binder post-treatments to enhance their mechanical properties. According to Ashby’s chart comparison, some of the produced materials already possess natural leather properties, whereas others are closely related.

### Bread waste hydrolysis, fungal cultivation, and hydrogel preparation from fungal cell wall materials

*Rhizopus delemar* is a fast-growing filamentous fungus chosen for this study for several reasons, such as its ability to grow on minimal nutrients^12^, natural synthesis of chitin and chitosan in the cell wall^15^, safety to work as a biosafety level 1 (BSL-1) strain (https://wi.knaw.nl/page/fungal_display/92976), and its ability to produce leather-like material^11^. The bread hydrolysate obtained from bread hydrolysis with alpha-amylase was used as a substrate to avoid entangling unconsumed bread particles in the fungal biomass. Thus, the particle-free fungal biomass was harvested after washing. The hydrolysate collected from the 20% w/w bread hydrolysis contained 14.7 ± 0.5% soluble solids. From 1000 l of 4% diluted bread hydrolysate substrate, 25 kg wet fungal biomass (ca. 11.5% dry weight) were collected. This led to a fungal biomass yield of 2.9 g/l or 0.072 g/g substrate. This yield is almost half of the yield (0.15 g/l) obtained from *R. delemar* cultivation in 4% bread powder suspension in water^11^ however, this higher yield could be partly due to the entanglement of unconsumed bread particles. Furthermore, obtaining clean biomass helped to obtain a clean final product made of fungi.

The fungal biomass was subjected to alkali treatment to extract the cell wall fraction. The filtered and washed solid residues appeared clumped after the treatment. The uniformity of the sheets can be affected if this clumpy material is used. To alleviate this, the fibrous cell wall material was converted into a hydrogel by adding dilute lactic acid to decrease the pH to 3^22^. After the addition of lactic acid and when the pH reached 3, the material turned into a hydrogel. This can be explained by protonation of the amino groups in chitosan available in the fungal cell wall by acid addition^22^. This hydrogel was used to prepare wet-laid sheets without further processing, as the goal was to use it as an inner reinforcement layer.

### Tanning of fungal biomass

**Fig. 2:**
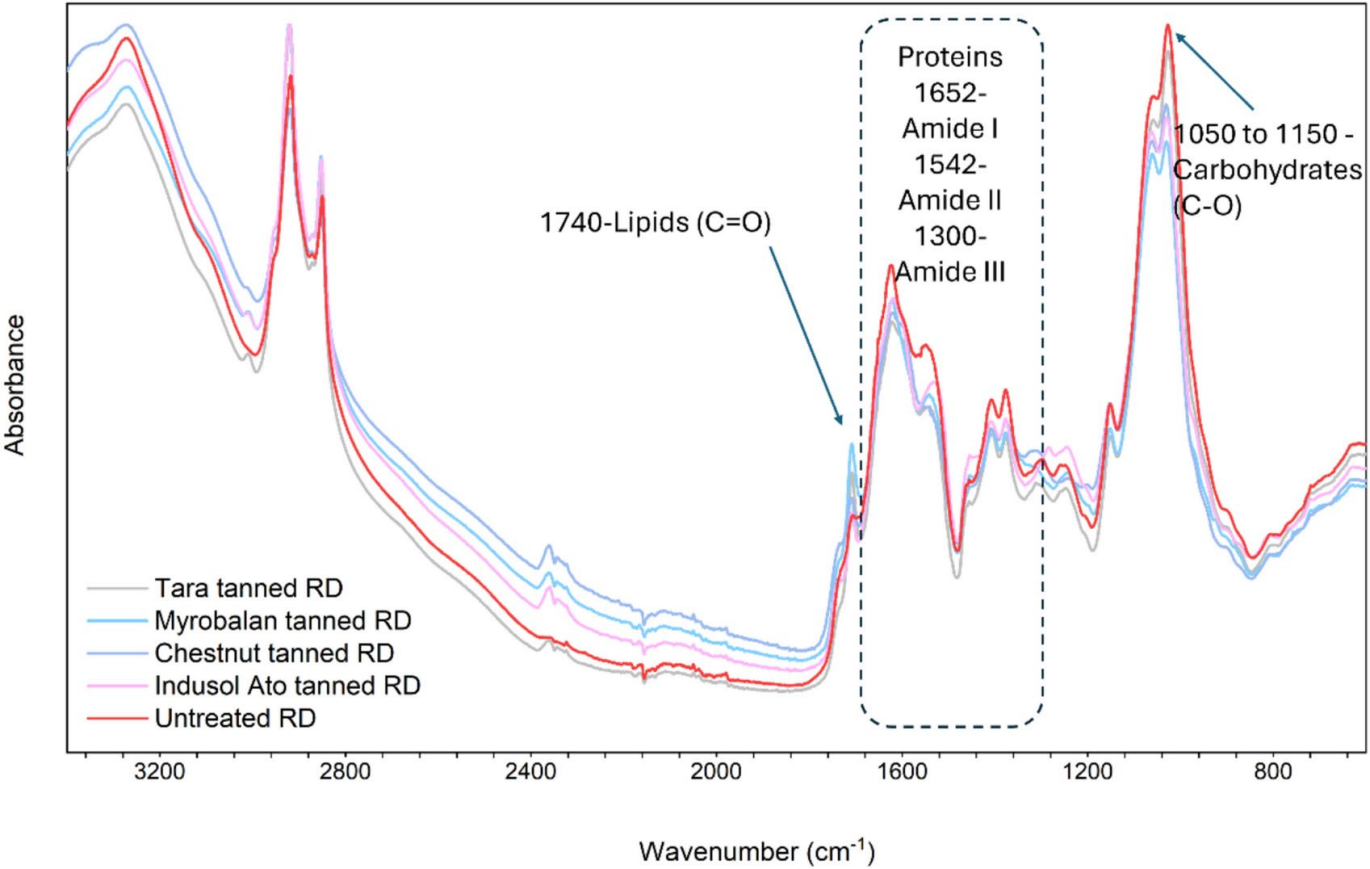
FTIR spectra of untreated biomass and different tannin-treated biomass.

Tanning, by definition, is a process which converts animal hides into leather^26,27^ using plant polyphenolic compounds known as tannins. In the tanning process, the reactions between the proteins available in animal hides and tannins are vital. These reactions occur via hydrogen bonding and hydrophobic interactions^28^. In our previous study, reactions between tannins from chestnut wood and fungal proteins via hydrophobic interactions and hydrogen bonds were explained using Fourier transform infrared spectrophotometry (FTIR) and nuclear magnetic resonance spectroscopy (^13^C NMR)^11^. In the current study, three extra tannin types (Tara, Myrobalan, and Indusol ATO), along with chestnut, were used. The tanning receipt described in our previous study^11^ was used to assess the effects of different tannins on the particle-free fungal biomass. By analysing the FTIR spectra (Fig. 2) of the tanned fungal biomass and untreated fungal biomass, the reaction between tannins and fungal proteins was confirmed. Generally, the peaks related to proteins between wavenumbers 1300 and 1700 cm^−1^^29^ shows variation in absorbance in tannin-treated fungal biomass spectra compared to the untreated biomass spectrum. More specifically, the amide II peak at 1542 cm^−1^^30^ shifted in all tanned samples compared to the untreated biomass sample. The peaks related to carbohydrates between 1050 and 1150 cm^−1^^30^ showed variations in absorbance in all tannin-treated samples. These findings confirm that all four different tannin types used have an affiliation with proteins and carbohydrates in fungal biomass^31^.

**Fig. 3:**
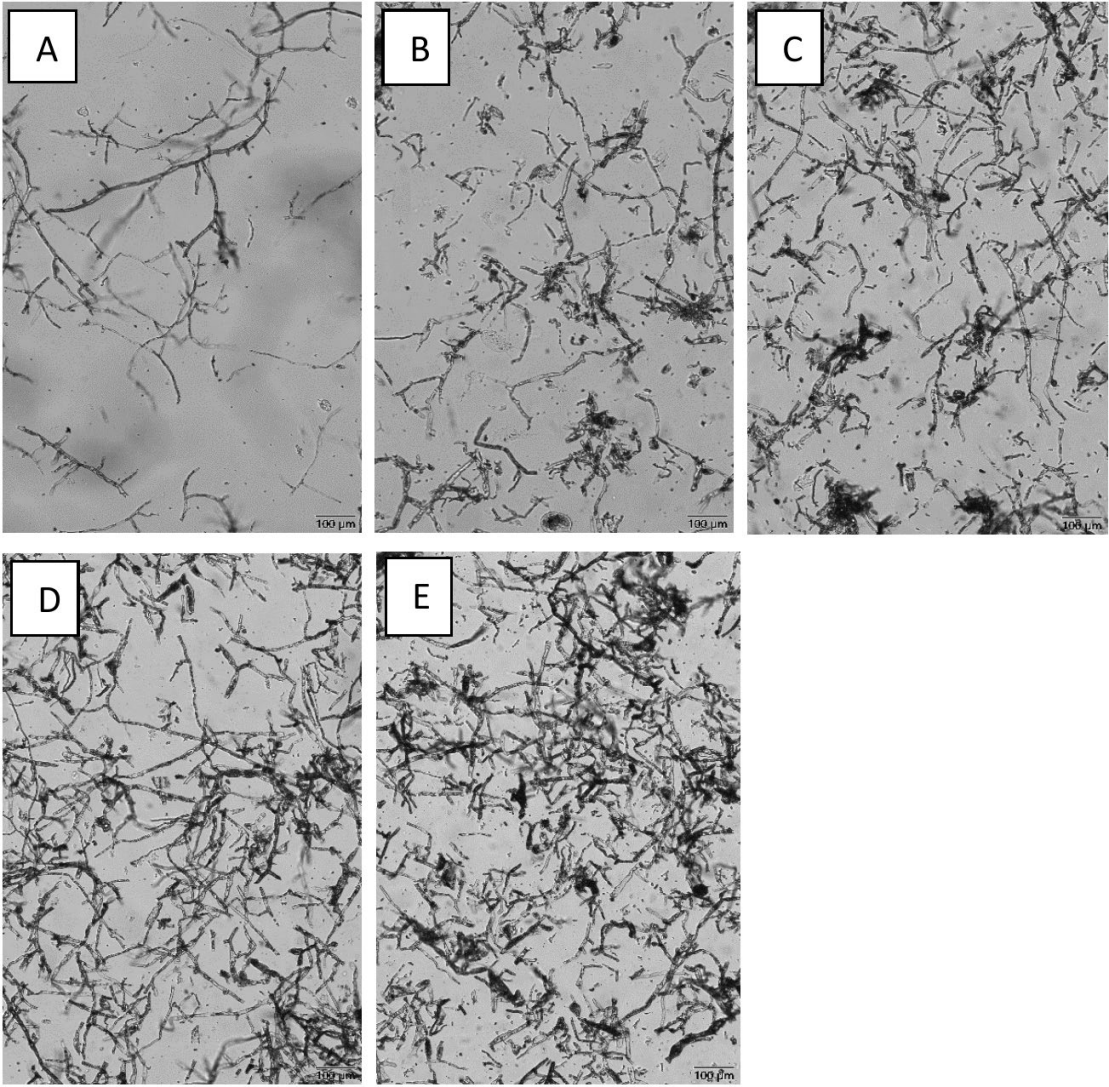
Microscopy images collected from oCelloScope (**A** untreated, **B** Tara, **C** Myrobalan, **D** Chestnut, and **E** Indusol ATO).

When the microscopic images were analysed, the swelling behaviour of the tannin-treated biomass and enlargement of the fungal hyphae after each tannin treatment were observed (Fig. 3). The average diameters of the hyphae for different fungal biomass treatments are listed in Table 2. This observation is similar to that reported in our previous work^11^ and further affirms the reaction of tannins with fungal biomass.

### Development of multilayer biocomposites to make vegan leather

The natural leather cross-section is composed of collagen fibres arranged in layers with different tightness gradients^7^. These collagen fibres become distinctive layers of separated fibrous sheets due to the removal of non-collagen components during the conversion of animal hides to leather^32^. We hypothesised that mimicking the multilayer arrangement of natural leather using different fungal materials would yield a composite material with enhanced properties compared with a monolayer material^33,34^. In our previous study, single-layer sheets exhibiting leather-like properties were produced from tanned fungal biomass using a wet-laying technique^11^. In addition, Benedikt Maria Köhnlein et al.^18^ isolated the chitin and chitosan-rich cell wall fraction from *R. delemar* and used it to produce sheets by wet-laying. In this study, both the tanned fungal biomass and cell wall fractions were used to prepare multilayer bio-composite sheets.

The sheets of tanned biomass were easy to remove from the vacuum funnel setup and were brittle when dried. The chestnut-tanned single-layer sheets showed a tensile strength and elongation % at break of 9.1 MPa and 3.5%, respectively (Table 3). These values were similar to those of the single sheets presented in our previous article^11^. Of the other three tannins, Tara and Myrobalan single-layer sheets showed higher tensile strengths, and Indusol ATO had a slightly lower tensile strength.

**Table 3:** Mechanical properties of the produced fungal sheets.

Sample	Tensile strength (MPa)	Elongation % at break	Young’s modulus (GPa)
Tara S	12.4 ± 1.0	2.6 ± 0.4	0.53 ± 0.21
Tara C	20.5 ± 0.3	7.2 ± 1.1	0.91 ± 0.22
Tara C G	12.6 ± 0.5	7.9 ± 1.2	0.65 ± 0.16
Tara C GB	6.9 ± 0.2	13.9 ± 1.6	0.35 ± 0.04
Myrobalan S	11.4 ± 0.7	3.4 ± 0.3	0.56 ± 0.02
Myrobalan C	15.1 ± 1.9	6.0 ± 1.1	0.62 ± 0.19
Myrobalan C G	8.1 ± 0.5	11.1 ± 0.8	0.39 ± 0.02
Myrobalan C GB	6.3 ± 0.6	14.8 ± 1.0	0.31 ± 0.02
Chestnut S	9.1 ± 1.2	3.5 ± 1.0	0.51 ± 0.19
Chestnut C	16.0 ± 0.9	6.6 ± 0.6	0.80 ± 0.05
Chestnut C G	9.7 ± 0.8	8.0 ± 0.3	0.51 ± 0.05
Chestnut C GB	8.0 ± 0.0	12.4 ± 0.2	0.41 ± 0.05
Indusol ATO S	8.3 ± 1.2	2.6 ± 0.5	0.49 ± 0.12
Indusol ATO C	19.0 ± 0.8	6.6 ± 0.5	0.87 ± 0.17
Indusol ATO C G	7.9 ± 0.3	10.1 ± 0.4	0.43 ± 0.05
Indusol ATO C GB	6.8 ± 0.4	11.5 ± 0.6	0.31 ± 0.05

**Fig. 4:**
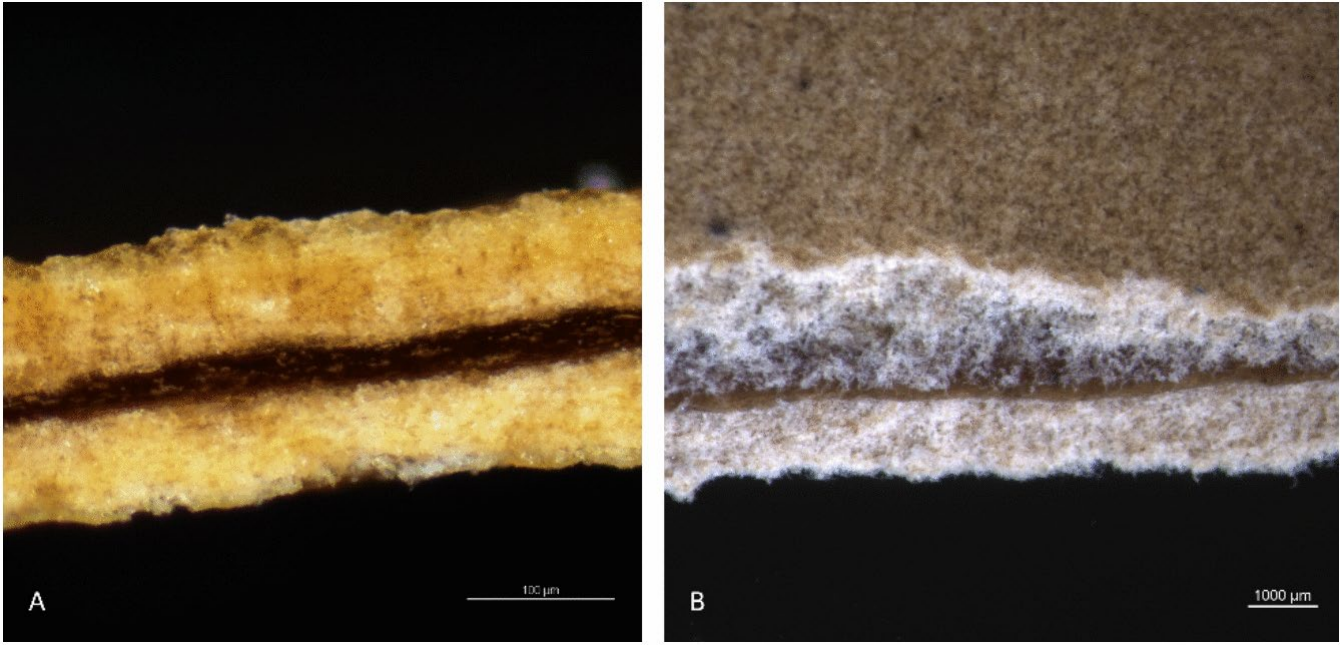
Optical microscopy cross-sectional images showing the three-layer sandwich structure (**A** direct cross-sectional view, **B** cross-sectional view from top with slant cut edge). Obtained from Nikon Eclipse LV150NL—Transmission Microscope.

Compared to the sheets made with tanned biomass, the sheets made from cell wall material were slightly harder to remove, owing to the gel-like behaviour of the material; thus, these sheets were unable to dewater with blotting papers. A multilayered composite arrangement was done when all the sheets were wet. Optical microscopy revealed a multilayered sandwich arrangement (Fig. 4). The surface became even once the biocomposites were pressed using a bench press. The composite sheets showed approximately 65%, 30%, 70%, and 130% increments in tensile strength in the Tara, Myrobalan, Chestnut, and Indusol ATO composite sheets, respectively, whereas the percentage of elongation at break increased by approximately two-fold in all cases (Table 3). The composites made with Tara and Indusol ATO tanned biomass showed significantly higher tensile strengths of 20.5 and 19.0 MPa, than their respective single-layered sheets which are 12.4 and 8.3 MPa (P value < 0.000). Those values are higher than the tanned sheets made in our previous work (9.6 MPa)^11^, vegan leather made by Akhter et al.^35^ using Jute, mycelium and polyhydroxyalkanoates (PHA), and many commercial alternative leathers such as Muskin® (0.2  MPa), Pinatex® (4.5 MPa) and Appleskin® (14 MPa) tested by Meyer et al.^7^.

The biocomposites post-treated with glycerol were softer in texture and more flexible. However, a considerable amount of tensile strength was lost because of the plasticising effect of glycerol. Myrobalan composite sheets after glycerol post-treatment (Myrobalan C G) showed the highest increase in elongation at break, from 6.0 to 11.1%. The bio-based binder post-treatment further increased the elongation % at break and reduced the tensile strength, thus increasing the flexibility and ductility of the sheets. Overall, Myrobalan composites after glycerol and bio-based binder post-treatments (Myrobalan C GB) showed the highest flexibility of 14.8% elongation at break. Although all the samples showed similar ductile behaviour, Tara and Myrobalan treated composites (Tara C GB and Myrobalan C GB) showed higher roughness on the surface compared to Chestnut and Indusol ATO sheets, thus with an intention to have a softer/better hand feel, Chestnut C GB and Indusol ATO C GB were used to prepare the prototype presented in this article.

**Fig. 5:**
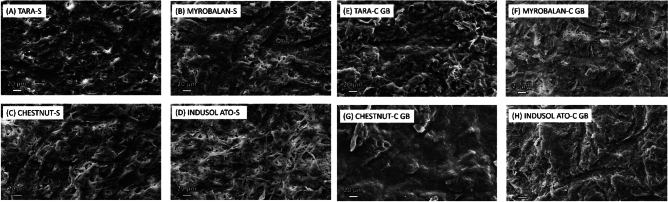
Scanning electron microscope (SEM) micrographs of the produced fungal sheets (*S* Single sheet, *C GB* Composites after glycerol and bio-based binder post-treatments).

Scanning electron microscopy (SEM) micrographs explain the surface morphologies of the produced biocomposite vegan leather materials. The SEM micrographs of single-layer sheets (Fig. 5AD) show a porous structure owing to the homogeneous spread of fungal microfibres during the wet laying process. The porous structure of sheets prepared with tanned biomass was also observed in our previous research^11^. Of the four tannin-treated composite SEM micrographs (Fig. 5E–H), the Chestnut—(Fig. 5G) and Indusol ATO—(Fig. 5H) treated composites had a more uniform surface morphology than the Tara- and Myrobalan-treated composites. This further confirms the softer hand feel of Chestnut- and Indusol-Ato-treated biocomposites that were chosen to prepare the prototype.

**Fig. 6:**
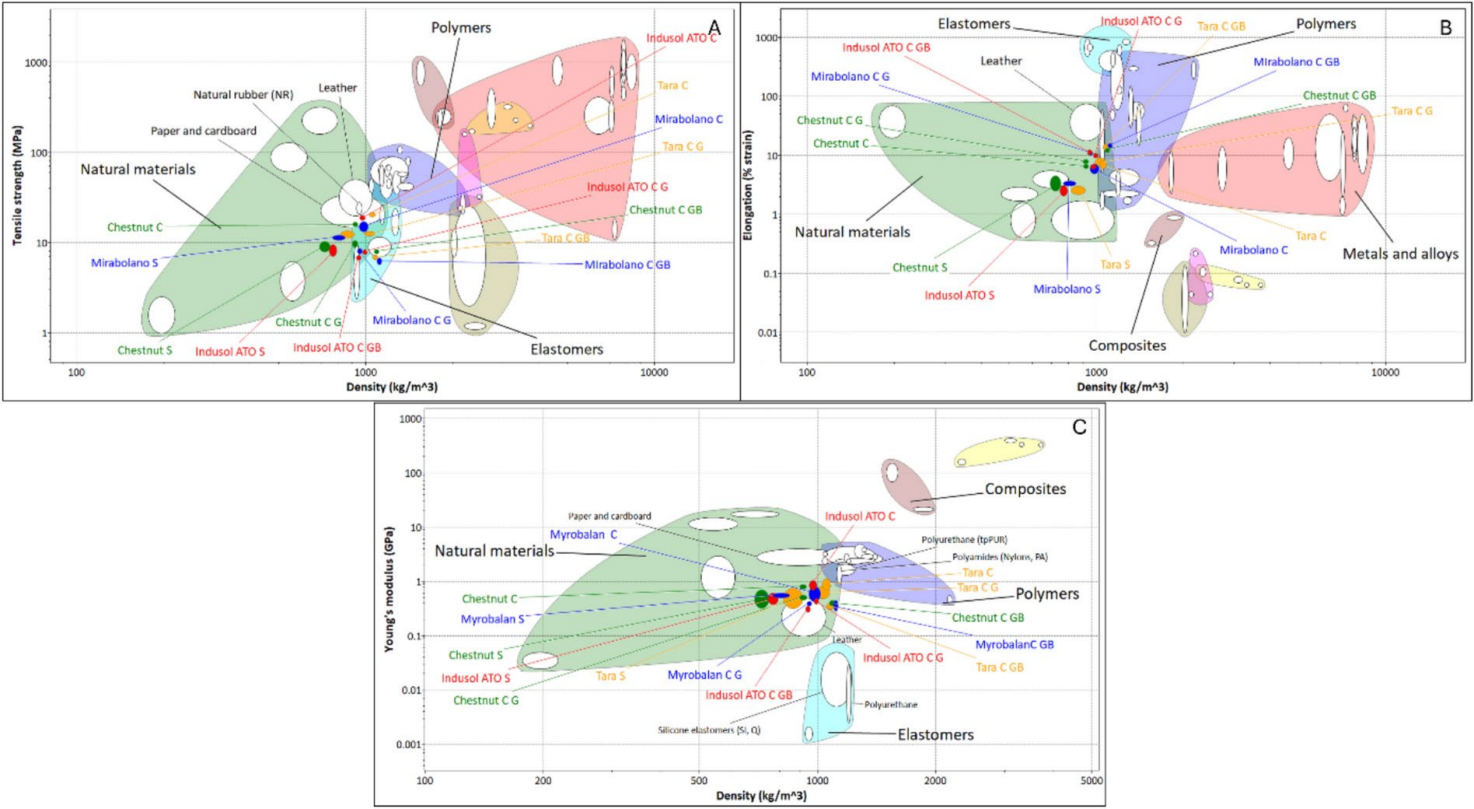
Ashby’s material property chart comparing the vegan leather sheets produced with natural leather. (**A**) Tensile strength vs density, (**B**) Elongation vs density, (**C**) Young’s modulus vs density. (Plotted using Granta EduPack 2021 R2 Version: 21.2.0).

Ashby’s material property charts in Fig. 6A–C were plotted to compare the fungal biocomposite sheets with the natural leather on tensile strength, elongation and Young’s modulus vs density in each graph. The main classes of materials, such as natural materials, polymers, elastomers, and composites, were included in the plot to observe the position of fungal sheets in the material universe. Almost all the fungal biocomposite vegan leather sheets are placed in the natural material bubble in all three material property comparisons. In the Fig. 6A, all the composite sheets (Tara C, Myrobalan C, Chestnut C, and Indusol ATO C) shows closer relationship towards natural leather owing the higher tensile strength of the composite sheets. However, with the plasticising post-treatments with glycerol and bio-based binder the tensile strength decreased and the three post-treated samples namely Tara C GB, Myrobalan C GB and Chestnut C GB sheets have moved down to elastomers bubble. In the Ansys database the tensile strength of natural leather is between 20 and 50 MPa. Nonetheless different scientific experimental literature shows different tensile strengths such as, 9.88 to 10.6 MPa in cow leather^36^, 6.02 to 20.97 MPa in sheep leather^37^ and 22.84 to 25.24 MPa in chrome tanned leather^38^. The fungal biocomposite vegan leather materials discussed in this study exhibit tensile strength values that are more comparable to those reported in existing literature.

For fungal based leather materials elongation is the most difficult property to achive in comparison to natural leather. In the Fig. 6B, the composite sheets after glycerol and bio-based binder post-treatments have moved towards the natural leather bubble however more improvements are needed to achieve proper elongation compared to natural leather. The elongation % of natural leather according the Ansys database is between 18 and 75. But in experimental literature the elongation % values of natural leather are always around 47 to 69^37,38^. In commercial leather process, treatments wich as fat liquring adds considerable amount of elasticity to the animal hide by maintaing the collagen fiber structure without drying and sticking^39,40^. Such treatments like fat liquiring could improve elongation % of the fungal biocomposite materials in the future.

When Young’s moduli and densities are compared (Fig. 6C) all the composite sheets (Tara C, Myrobalan C, Chestnut C, and Indusol ATO C) deviated towards the higher side of the Young’s modulus axis, indicating that the composites are brittle compared to natural leather. After glycerol post-treatment, the composites were moved towards natural leather and the Myrobalan-tanned composite sheet (Myrobalan C G) was placed inside the leather bubble. After the bio-based binder application, all the composites except the Indusol ATO tanned sheet (Indusol ATO C GB) deviated towards the higher side of the density axis because of the higher weight gain from the post-treatment. Overall, Young’s modulus wise most of the produced biocomposite vegan leather sheets are more closer to natural leather. Further improvements scpecially in fexibility will increase the likeliness of these novel material towards natural leather.

### Lightfastness of the fungal vegan leather

**Fig. 7:**
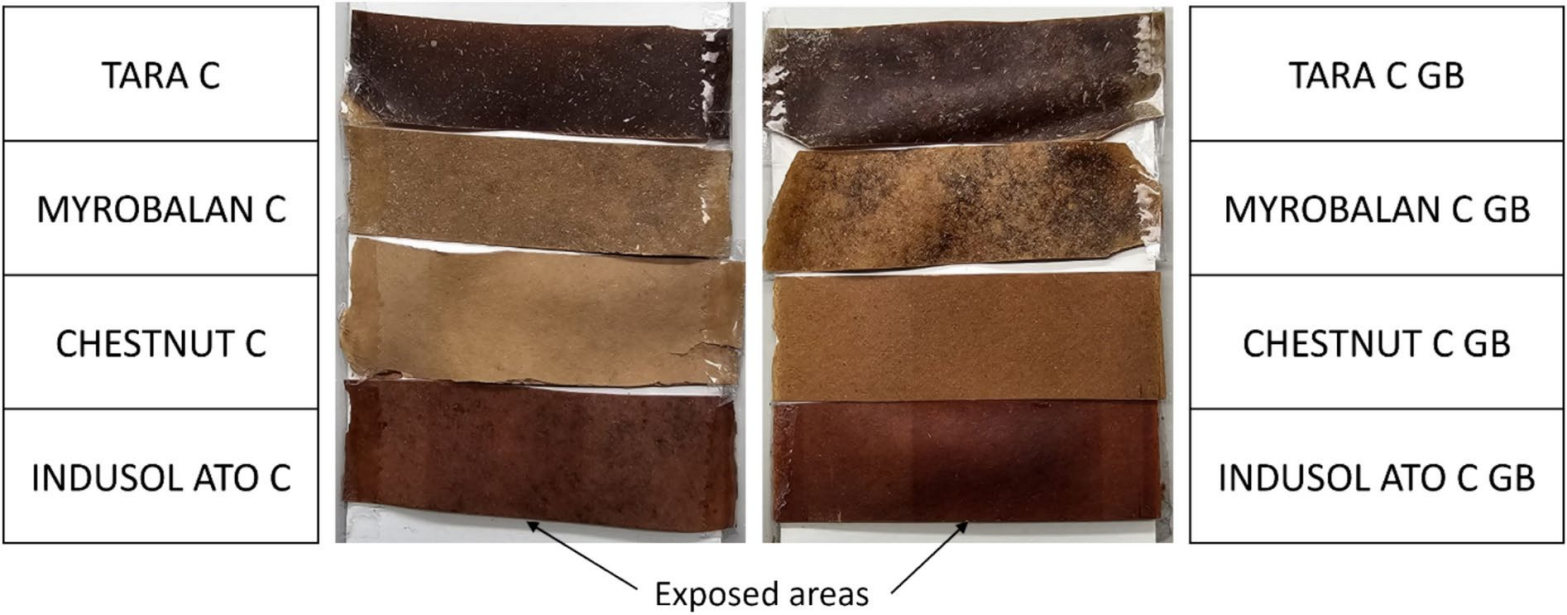
Samples from lightfastness test under D65 artificial daylight.

The light-fastness properties of the fungal biocomposites are shown in Table 4 and Fig. 7. In the table, the results are shown in grey scale ratings (GS_c_) from 1 to 5 where 1 is for most colour difference and 5 is for no colour difference. All tannin-treated composites, with and without glycerol and bio-based binder post-treatments, were tested. Untreated and post-treated Tara and Myrobalan-tanned composites showed little colour fading (3–4 or 4–5 GS_c_ in Table 4), while Chestnut and Indusol ATO-tanned composites became darker in both untreated and post-treated samples (Fig. 7).

**Table 4:** Lightfastness of the multilayer fungal biocomposites in grey scale ratings (GS_c_) obtained from Datcolour TOOLS Plus software.

Sample	Grey scale rating for colour change (GS_c_)
Tara C	3–4
Myrobalan C	3–4
Chestnut C	3–4
Indusol ATO C	3
Tara C GB	3–4
Myrobalan C GB	4–5
Chestnut C GB	3
Indusol ATO C GB	1–2

This darkness was measured using a spectrophotometre, and as shown in Table 4, these changes were 3–4, 3, 3 and 1–2 for Chestnut C, Indusol ATO C, Chestnut C GB, and Indusol ATO C GB, respectively. In their article Musa et al.^41^ have reported similar ratings in gray scale (3 and 3–4) when natural leather dyed with henna with in both pre and post dye fixing metal ions (mordants). The darkening of the colour in the exposed area of the sample was unexpected. This darkness could be due to the formation of quinones on the vegetable tannin phenolic structure^42^. The results further explain that within the first 24 h, the hydrolysable tannins such as Tara, Myrobalan, and Chestnut show lower colour differences, while condensed tannins such as Indusol ATO (Quebracho extracts) showed a higher colour change which is in line with our observations.

### Abrasion resistance (martindale method)

**Fig. 8:**
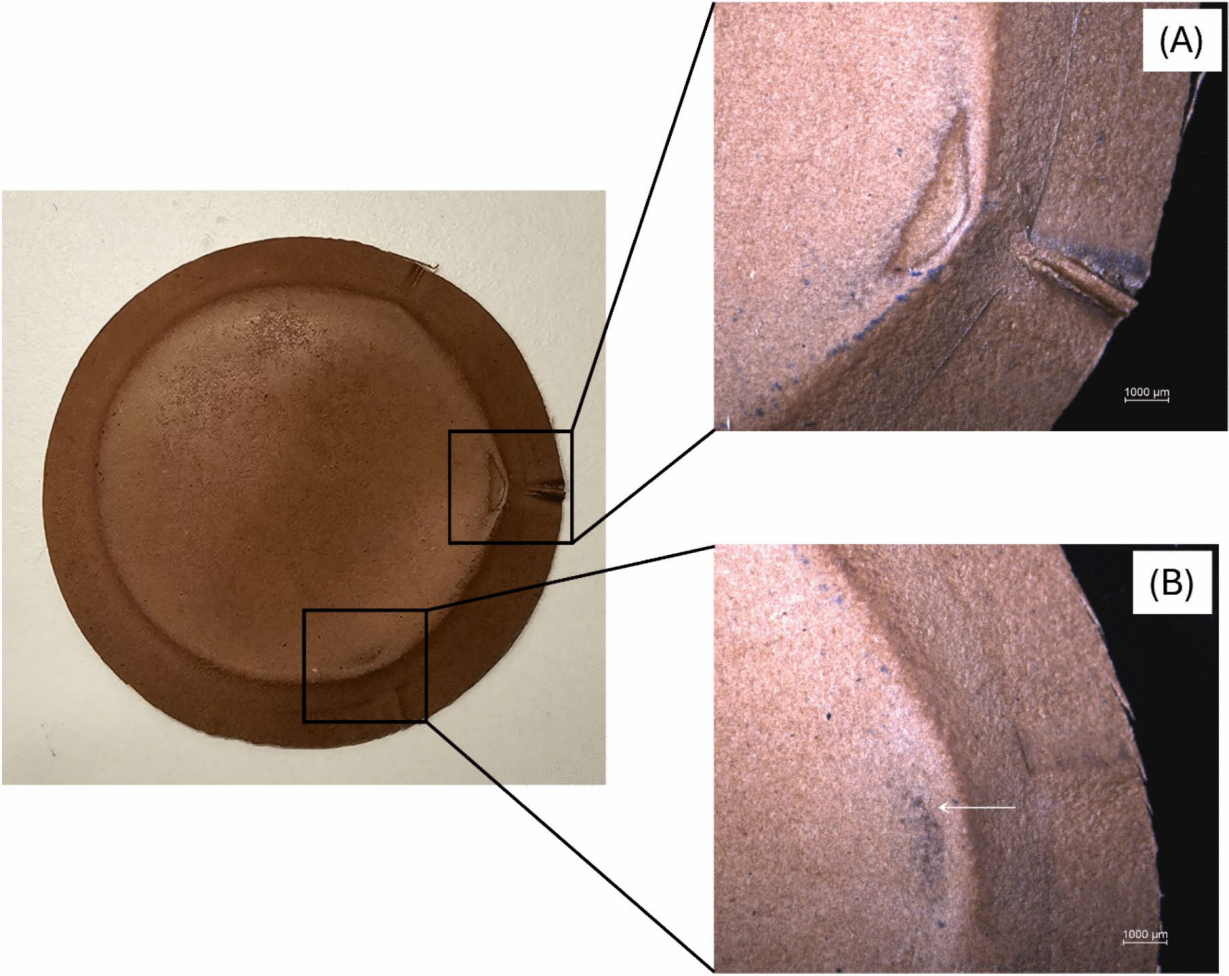
Two finish breakdown places of Indusol ATO C GB sample and their respective optical microscopic images (**A**, **B**).

The requirements for the abrasion resistance of leather are highly dependent on the application. Nevertheless, understanding the properties of a novel material would still be advantageous when possible future applications are discussed. The Martindale abrasion test was conducted for two selected fungal biocomposite samples, chestnut-tanned biomass and Indusol ATO-tanned biomass, after both glycerol and bio-based binder post-treatments (Chestnut C GB and Indusol ATO C GB) as they were chosen for further prototyping. After the initial 100 rubs, only one sample out of the Chestnut C GB replicates had one finish breakdown location, and only one sample out of the Indusol ATO C GB replicates had two finish breakdowns. After 3000 rubs, both Chestnut C GB replicates and one sample of Indusol ATO C GB had four finish breakdown places, and the remaining Indusol ATO C GB sample had only two finish breakdown places (Fig. 8). According to two published patents, the abrasion resistance values of cow, kangaroo, goat, python, and stingray are 185, 350, 750, 850, and 1000 + rubs respectively^43^. These values were obtained from another test method known as the Taber method which is a more rigorous method for testing leather abrasion resistance^44^. Even though results from Martindale method and Taber method are difficult to compare directly 3000 rubs from Martindale method shows acceptable level of positive results compared to the sub 1000 rubs from Taber method of commercial natural leathers.

### Development of a prototype from fungal vegan leather

**Fig. 9:**
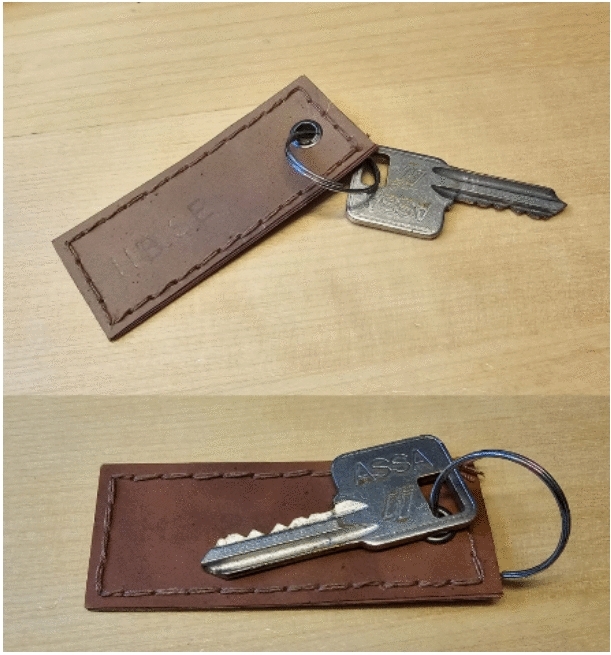
The prototype made using Chestnut (Chestnut C GB-top picture) and Indusol ATO (Indusol ATO C GB-bottom picture) composites after glycerol and bio-based binder post-treatments (on either sides).

Multilayer biocomposites made from chestnut-tanned biomass and Indusol ATO-tanned biomass after both glycerol and bio-based binder post-treatments (Chestnut C GB and Indusol ATO C GB) were used to prepare the prototype. A hanging ornament for the keyring was created by stitching two pieces from either side (Fig. 9). Stitching was easier because the two sheets were flexible. The ability to use a letter puncher is demonstrated; however, further improvements are needed for a perfect letter embossing effect. Hole punching was performed using a leather punching tool and the eyelet was fixed to reinforce the hole. Two-way stitching was performed to provide a better final product finish. Overall, the simple prototype demonstrated the workability of the novel bio-composite vegan leather made from fungi.

## Conclusion

The filamentous fungus *Rhizopus delemar* was successfully cultivated in a scaled-up process in a 1300 L pilot scale bubble column bioreactor. Four different commercial vegetable tannins were tested for the tanning of fungal biomass, and their reactions with fungal proteins and carbohydrates were demonstrated. Furthermore, the fibrous cell wall fraction which is rich in biopolymers, such as chitin and chitosan, was extracted from the fungal biomass using a mild alkali treatment. Fungal based biocomposites were successfully produced using both tanned biomass and the fibrous cell wall fraction and tested as vegan alternative leather material. Fungal sheets and composites were prepared using wet-laying technique, a common method used in paper production. The produced fungal biocomposites have properties comparable to those of natural leather, and the workability of the materials is demonstrated using a simple prototype. Overall, this article shows the potential of material bio-fabrication using fungi as a vegan alternative leather (Supplmentary information).

## Supplementary Information

Supplementary Information.

## Data Availability

The datasets generated and/or analysed during the current study are available from the corresponding authors upon reasonable request.
